# Impact of coping with interpersonal stress on the risk of depression in a Japanese sample: a focus on reassessing coping

**DOI:** 10.1186/s40064-015-1111-7

**Published:** 2015-07-04

**Authors:** Tsukasa Kato

**Affiliations:** Department of Social Psychology, Toyo University, Hakusan 5-20-26, Bunkyo-ku, Tokyo, Japan

**Keywords:** Coping behavior, Interpersonal stress coping, Depression, Prevalence, Reassessing coping

## Abstract

Reassessing coping, a concept first proposed in Asia, refers to efforts to wait patiently for an appropriate opportunity to act or for a change or improvement in a situation, and can be observed in individuals facing stressful relationship events. The main purpose of the present study was to determine if reassessing coping would be associated with a lower risk of depression. The author examined the relationships between depression risk using the Center for Epidemiologic Studies Depression Scale (CES-D) and coping strategies for interpersonal stressors, including reassessing coping, in a sample of 1,912 Japanese college students. In our sample, the proportions of women and men with depressive symptoms were 55.28% (95% confidence intervals (CIs) [52.35, 58.20]) and 46.08% (95% CIs [42.63, 49.52]), respectively, using the conventional CES-D cut-off score of 16. A multivariate logistic regression analysis revealed that reassessing coping was significantly associated with a lower risk of depression (OR = 0.92, 95% CIs [0.89, 0.95]), after adjusting for gender. Distancing coping (strategies to actively damage, disrupt, or dissolve a stressful relationship) and constructive coping (strategies to improve, maintain, or sustain a relationship without irritating others) were significantly associated with a greater depression risk. Reassessing coping for interpersonal stressors was associated with a low risk of depression among Japanese college students.

**Electronic supplementary material:**

The online version of this article (doi:10.1186/s40064-015-1111-7) contains supplementary material, which is available to authorized users.

## Background

Depression is a leading cause of disability worldwide. According to face-to-face household surveys of 60,463 community-dwelling adults by the World Health Organization (WHO 2004), the prevalence of mood disorders ranged from 0.8% in Nigeria to 9.6% in the United States. Furthermore, analyses by the WHO (2001) revealed that among individuals aged 15–44 years worldwide, unipolar depressive disorders were the leading causes of burden among all disease, accounting for 8.6% of total disability-adjusted life years (DALYs) and 16.4% of years of life lived with disability (YLDs). DALYs are calculated as the sum of the years of life lost due to premature mortality in the population and the YLD for people living with the health condition or its consequences (WHO 2001).

Given the considerable burden of depression, it is necessary to examine factors contributing to the risk or recurrence of depression. One such factors interpersonal stressors. Several researchers (e.g., Hames and Shin 2014; Hankin and Abramson 2001; Joiner and Coyne 1999; Rudolph 2009) have proposed mechanisms by which interpersonal stressors increase the risk and recurrence of depression, and have provided evidence for the relationships between interpersonal stressors and depression. For example, Vrshek-Schallhorn et al. (2014) found a gene environment interaction effect between the serotonin transporter-linked polymorphic region (5-HTTLPR) and interpersonal stressful events on risk of major depressive episode onset as assessed by the Structured Clinical Interview for DSM-IV Axis I Disorders; in contrast, non-interpersonal stressful events showed no such interaction effect. According to theories of depression vulnerability, interpersonal stressful events trigger depressive episodes and the development of depression, particularly for individuals with interpersonal vulnerability (Hankin and Abramson 2001; Joiner and Coyne 1999; Rudolph 2009). This suggests that people of Asian cultures would be more susceptible to the effects of interpersonal stress, given that Asian cultures (including Japanese culture) typically have social values based on collectivism and interdependence, in contrast to the Western cultural values of individualism and independence. Furthermore, Asian cultures emphasize respecting others, living in harmony, and basing one’s behavior on the perceived thoughts, feelings, and actions of others (Markus and Kitayama 1991; Triandis 1995). Given this emphasis, it is especially important to identify the various coping strategies that Asian people, and specifically Japanese, have for dealing with interpersonal stressors, as it would contribute to reducing the risk of depression in these countries.

The WHO (2001) has stated that certain of types of mental and behavioral disorders, such as depression and anxiety, can occur as a result of failing to cope adaptively with a stressor. Indeed, according to the transactional theory proposed by Lazarus and his colleagues (Lazarus 1999; Lazarus and Folkman 1984), coping behavior defined as “constantly changing cognitive and behavioral efforts to manage specific external and/or internal demands that are appraised as taxing or exceeding the resources of the person” (p. 141, Lazarus 1999) affects psychological functioning, including depressive symptoms. The transactional theory has been supported by numerous studies (for reviews, see Lazarus 1999; Lazarus and Folkman 1984).

Drawing on transactional theory, Kato (2013) proposed *reassessing coping*, which refers to coping strategies for interpersonal stress that may reduce the risk of depression. More specifically, reassessing coping refers to waiting patiently for an appropriate opportunity to act or for a change or improvement in a situation. Kato (2013) stated that reassessing coping provides people with the time necessary to manage their stressful relationships, better understand situations they encounter, control their emotions, and evaluate different plans of action. Reassessing coping may also influence other individuals involved within a stressful relationship, helping to change the other party’s mental state and thus permitting them to adopt a calmer, more accepting attitude towards the stressful relationship. As a result, reassessing coping increases the likelihood that the situation will improve. Stressful relationships constantly change. For example, immediately after having a quarrel, people involved in a stressful relationship may be excited; however, they may recover their mental balance over time. In such cases, a strategy that involves waiting to deal with the stressful relationship until the people involved have recovered mental balance may produce adaptive outcomes for individuals experiencing stressful events. Therefore, reassessing coping enables people to select an appropriate strategy, at an adaptive time, according to changing stressful relationships. In other words, reassessing coping helps individuals manage stressful relationships in flexible ways that might work to improve the situation (Kato 2014a, b). Research on flexibility, especially coping flexibility, has highlighted these strategies as important processes allowing individuals to flexibly cope with stressful situations (for reviews, see Bonanno and Burton 2013; Cheng et al. 2014; Kato 2012).

Reassessing coping is distinct from avoidant coping, which involves avoiding stressful problems or situations (e.g., wishful thinking and behavioral efforts to escape or avoid the problem). Instead, it involves active strategies requiring self-control and restriction of premature action. Indeed, a previous study (Kato 2013) found a non-significant negative correlation between reassessing and avoidant coping (*r* = −11, *N* = 184) and significant positive correlations between reassessing and active strategies (*r*s = 0.39 and 0.37, *N* = 184), such as restraint coping (holding oneself back, not acting prematurely, and waiting until an appropriate opportunity arises) and detached coping (a feeling of emotional distance from stressful events).

Several studies have demonstrated that reassessing coping can reduce depressive symptoms resulting from stressful relationship events. For example, when using reassessing coping, full-time Japanese employees showed reductions in levels of depressive symptoms resulting from interpersonal stress in the workplace (Kato 2014a). In addition, 424 Japanese teachers reported that reassessing coping was associated with lower levels of depressive symptoms (Taniguchi and Tanaka 2014). Correlations between reassessing coping and reduced depressive symptoms have been reported in studies with Japanese college student samples (Kato 2013) as well as with the American, Australian, and Chinese general populations (Kato 2014b).

The purpose of the present study was to assess the relationship between depression risk using the Center for Epidemiologic Studies Depression Scale (CES-D) and coping strategies (specifically reassessing coping) in a large sample of Japanese college students. We hypothesized that reassessing coping would be associated with a low risk of depression using the CES-D.

## Methods

### Participants and procedure

Participants were 1,912 Japanese undergraduates (1,109 women and 803 men; *M* = 19.56, SD = 1.34 for age) enrolled in introductory psychology classes at five colleges or universities in the Kanto Region. Their majors were literature or sociology including psychology (31%), law or political science (26%), economics or business administration (22%), and science or engineering (13%). All participants had been born in Japan and noted their ethnicity as Japanese. After giving informed consent, they completed a set of questionnaires to assess coping with interpersonal stress and depression risk using the CES-D. All participants were informed orally and in writing about the following: the purpose of this study, the contents of the questionnaire, their right to decline or withdraw participation, their confidentiality, etc. Participants did not receive any compensation for their participation.

### Measures

#### Reassessing coping

The Interpersonal Stress Coping Scale (ISCS; Kato 2013) was used to measure coping with interpersonal stressors (see Additional file 1). The ISCS consists of three 5-item subscales evaluating reassessing coping (e.g., taking a pragmatic view of the matter, deciding not to take the matter too seriously), distancing coping (e.g., avoiding contact with the person, ignoring the person), and constructive coping (e.g., reflecting on one’s own conduct, trying to understand the other person’s feelings). Distancing coping reflects strategies to actively damage, disrupt, and dissolve a stressful relationship, that is, distancing in this coping style means intentionally breaking off relations with another individual involved within the stressful relationship. On the other hand, constructive coping involves actively seeking to improve, maintain, or sustain a relationship without irritating others, and for which mutual respect and living in harmony are emphasized. Constructive coping includes several characteristics of collectivistic cultures (Kato 2013) as they emphasize respecting and living in harmony with others, and how one behaves is often based on perceptions of the thoughts, feelings, and actions of others (Triandis 1995). In fact, a cross-cultural study (Kato 2013) suggested people in collectivistic cultures, such as Japan, constructive coping use more frequently than do those in other countries.

The scores for the three strategies are associated with theoretically related constructs, such as other coping strategies and personality traits (Kato 2013). For example, reassessing coping is significantly associated with restraint and detached coping (Kato 2013); restraint coping is, like reassessing coping, an active strategy involving holding oneself back, not acting prematurely, and waiting until an appropriate opportunity arises; detached coping refers to attempting to gain a sense of emotional distance from stressful events. The Cronbach’s alphas for scores on reassessing, distancing, and constructive coping were 0.81 (95% confidence intervals (CIs) [0.78, 0.83]), 0.84 (95% CIs [0.82, 0.85]), and 0.73 (95% CIs [0.70, 0.76]), respectively (Kato 2013); these alphas were calculated using eight samples (*N* = 3,686).

The instructions on the ISCS were as follows: “Please recall the specifics of your own experiences of stress due to interpersonal relationships. These may include quarreling with others, being talked about behind your back, feeling awkward while speaking, and worrying if you have hurt someone’s feelings. Please read each item and indicate to what extent you used that strategy in the situations you encountered.” For each item, participants were asked to rate the extent to which they used each strategy to deal with interpersonal stressors on a 4-point Likert scale ranging from 0 (*did not use*) to 3 (*used a great deal*).

#### Depression risk

The Center for Epidemiologic Studies Depression Scale (CES-D; Radloff 1977), a 20-item self-report scale, was used to estimate the depression risk in this study (see Additional file 1). In a Japanese sample, a CES-D cutoff of 16 yielded a sensitivity of 0.95 (95% CI [0.89, 0.98]) and specificity of 0.29 (95% CI [0.23, 0.36]) for major depressive disorder (Furukawa et al. 1997). The CES-D was originally developed using data from the general American population (Radloff 1977); however, the validity and reliability of CES-D scores have also been established in the Japanese population (Furukawa et al. 1997; Shima 1998). Participants rated each item according to their experiences within the past week on a 4-point Likert scale ranging from 0 (*rarely or none of the time, less than 1* *day*) to 3 (*most or all of the time, 5*–*7* *days*). A CES-D score of 16, a traditional cut-off point recommended for Japanese samples (Shima 1998), was used to identify which participants had a likelihood of developing depression.

### Data analysis

A multivariate logistic regression analysis was conducted to compute the adjusted odds ratios (ORs) associated with depressive symptoms. The prevalence of depression was computed with 95% CIs. Although we focused on reassessing coping in the present study, distancing coping and constructive coping were also entered as predictors into the regression analysis to control for their effects on depression risk. Statistical analyses were performed using SPSS version 22 and R version 3.0.2.

## Results

Means, standard deviations, and Cronbach’s alphas for all variables are presented in Table 1, and zero-order and partial correlations are shown in Table 2. The mean scores for the CES-D for women and men were 18.86 and 17.53, respectively. The proportions of women and men with depressive symptoms (using a CES-D score of 16 as the cut-off) were 55.28% (95% CIs [52.35, 58.20]) and 46.08% (95% CIs [42.63, 49.52]), respectively; thus, the prevalence in women was higher than that in men (*B* = 0.37, SE = 0.09, Wald = 15.73, OR = 1.45, *p* < 0.001, 95% CIs [1.21, 1.74]).

**Table 1:** Means, standard deviations (SDs), and alphas for all variables

Value	Men (*n* = 803)		Women (*n* = 1,109)		*t* value	*p* value	Alpha
	Mean	SD	Mean	SD			
Reassessing coping	7.85	3.22	7.76	3.34	0.60	0.546	0.81
Distancing coping	3.50	3.41	3.13	3.00	2.51	0.012	0.75
Constructive coping	7.03	3.01	6.88	2.98	1.13	0.257	0.65
Depressive symptoms	17.53	10.69	18.86	10.02	2.77	0.006	0.87

**Table 2:** Zero-order correlations for all values and partial correlations between coping strategies and depressive symptoms

	Value	Zero-order correlation			Partial correlation
		2	3	4	4
1	Reassessing coping	0.13***	−0.02	−0.12***	−0.16***
2	Distancing coping		−0.15***	0.22***	0.27***
3	Constructive coping			0.17***	0.21***
4	Depressive symptoms				

*** *p* < 0.001.

The multivariate logistic regression analysis revealed that reassessing coping scores were significantly associated with a lower risk of depression (OR = 0.92, 95% CIs [0.89, 0.95], *p* < 0.001), after adjusting for gender. In contrast, distancing (OR = 1.16, 95% CIs [1.13, 1.20], *p* < 0.001) and constructive (OR = 1.12, 95% CIs [1.08, 1.16], *p* < 0.001) coping scores were significantly associated with a higher risk of depression, after adjusting for gender (Table 3).

**Table 3:** Risk factors of depression

Risk factor	B	SE	Wald	OR	95% CI	
					LL	UL
Gender						
Men				1.00		
Women	0.46	0.10	22.52	1.59***	1.31	1.92
Coping Strategy						
Reassessing coping	−0.08	0.01	31.50	0.92***	0.89	0.95
Distancing coping	0.15	0.02	85.68	1.16***	1.13	1.20
Constructive coping	0.11	0.02	47.27	1.12***	1.08	1.16

*OR* odds ratio, *CI* confidence interval, *LL* lower limit and *UL* upper limit. *** *p* < 0.001.

## Discussion

We aimed to investigate the relation between reassessing coping and depressive symptoms in a sample of Japanese college students. As expected, reassessing coping was significantly related with a lower risk of depression compared to other coping styles.

The CES-D scores of our sample may have been relatively high; however, CES-D scores in Japanese samples are often relatively high in comparison to samples from other countries. For example, the North West Adelaide Health Study (2007) reported that the prevalence of depressive symptoms for 3,057 Australian university students was 12.4%, using the same cut-off point on the CES-D as we did (i.e., 16). In contrast, a study of university students in Japan (Iwata and Buka 2002) showed a relatively high prevalence (52.2%, 95% CIs [46.5, 57.8]) of depressive symptoms and high mean CES-D scores (17.22, *SE* = 0.53), as in our study. Furthermore, our data on the gender differences in the depression risk were consistent with the literature that indicates that women experience depression more often than men do (for reviews, see Hilt and Nolen-Hoeksema 2014; Piccinelli and Wilkinson 2000).

With the exception of reassessing coping, no single strategy has been shown to be effective for coping with psychological distress (including depressive symptoms) resulting from interpersonal stress. In a meta-analytic review of 11 coping types, Penley et al. (2002) found that for interpersonal relationship stress, all coping styles were either negatively or not related to psychological distress (including depressive symptoms). Therefore, our findings may help in the reduction and prevention of depressive symptoms. However, there may be more effective strategies to reduce depressive symptoms in research areas other than coping for interpersonal stress.

Consistent with previous research in Japanese samples (e.g., Kato 2013, 2014a, c) and in other countries (e.g., Kato 2014b), distancing coping was significantly associated with a higher risk of depression. Kato (2014a) argued that distancing coping contributes to poor interpersonal relationships, which in turn can lead to increased depressive symptoms. Thus, reducing the use of distancing coping during stress management may help decrease the risk of depression.

Constructive coping was also significantly associated with a high risk of depression. This result was consistent with findings from a study (e.g., Kato 2014b) measuring constructive coping in American and Australian samples, and from a study (Seiffge-Krenke 2006) measuring strategies similar to constructive coping. However, the relations in other studies on this topic (e.g., Kato 2013, 2014a) were not significant. Individuals who frequently engage in constructive coping may value social interactions more highly than those who infrequently engage in it, because this strategy emphasizes respecting and living in harmony with others. As such, those who value social interactions to a greater degree may be more susceptible to the negative effects of interpersonal stress. Likewise, individuals with hypersensitivity to social interaction may frequently engage in constructive coping. Several studies (e.g., Gunthert et al. 2007; O’Neill et al. 2004) have suggested that hypersensitivity to social interaction predicts depressive symptoms for interpersonal stressful events, but not for non-interpersonal stressful events. To better elucidate the reasons for this negative relation between constructive coping and depressive symptoms, future studies should take into account other factors related to depressive symptoms.

Importantly, the sample size in the current study was far larger than that used in previous studies (e.g., Kato 2013, 2014a, b, c) on the relation between reassessing coping and depressive symptoms, and in previous studies (e.g., Furukawa et al. 1997; Iwata and Buka 2002) on the CES-D in Japanese college students. For example, Iwata and Buka’s study (2002), which is frequently cited by researchers as representative of CES-D scores in Japanese college students included only 310 participants. The CES-D has been widely used in many countries and with many racial/ethnic groups (Kim et al. 2011)—indeed, the article reporting on the development of the CES-D (Radloff 1977) was listed as 51st (with 17,055 citations) out of 100 in a list of the most-cited papers of all time by *Nature* (van Noorden et al. 2014) in 2014. This suggests that our data on the CES-D would be particularly useful for behavioral medicine research.

## Limitations

Despite the strengths of the study, several limitations warrant caution in the interpretation of the findings. First, a cross-sectional design using self-report measures was employed, meaning that a causal relationship between variables (coping with interpersonal stress and depression risk) cannot be inferred. For example, it is possible that being at risk for depression leads one to use specific coping strategies more often compared to those with no such risk. To make causal inferences, we would need to obtain further information from experimental studies with clinical and nonclinical populations.

Second, because this study included only a nonclinical sample of Japanese college students, the results may not generalize to other populations. Reassessing coping has been found to be associated with decreased depressive symptoms in the Chinese, Australian, and American general populations in previous research (Kato 2014b). However, further studies are needed to examine the relationships between coping strategies for interpersonal stress and depressive risk among other populations.

Finally, ORs of coping strategies for interpersonal stress, as a risk factor for depression might be relatively smaller, compared to other characteristics identified as risk factors for depression, such as younger age, longer duration of depressive episode, and a family history of mood disorders (for a review, see Hölzel et al. 2011).

## Conclusion

Despite the limitations of the present study, our results indicate that reassessing coping, a concept developed in an Asia population, is associated with a low risk of depression among Japanese college students. These findings extend current research regarding depressive symptoms.

## Additional file

**Additional file 1.** The interpersonal stress coping scale (Kato 2013) and the center for epidemiologic studies depression scale (Radloff 1977).
