# Supplementary material for: Impact of coping with interpersonal stress on the risk of depression in a Japanese sample: a focus on reassessing coping
Source: Springerplus. 2015 Jul 4;4:319. doi: 10.1186/s40064-015-1111-7 (PMC4491090; doi:10.1186/s40064-015-1111-7)
Supplement: Supplementary file 1 — Additional file 1. The interpersonal stress coping scale (Kato 2013) and the center for epidemiologic studies depression scale (Radloff 1977). [file 40064_2015_1111_MOESM1_ESM.docx]

Supplementary Table

The Interpersonal Stress Coping Scale (Kato, 2013)

|  |  |
| --- | --- |
| No. | Item |
|  |  |
| 1 | Tried to avoid talking with the person |
| 2 | Tried have only superficial associations with the person |
| 3 | Tried to talk it through thoroughly |
| 4 | Decided not to take the matter serious |
| 5 | Spent time as usual |
| 6 | Took a pragmatic view of the matter |
| 7 | Decided not to have anything more to do with the person |
| 8 | Tried not to worry about it |
| 9 | Tried hard to prevent the other person from feeling bad |
| 10 | Reflected on one’s own conduct |
| 11 | Examined and corrected one’s self |
| 12 | Tried to understand the other person’s feelings |
| 13 | Thought that a solution would be found somehow or other |
| 14 | Tried to ignore the person |
| 15 | Tried to avoid contact with the person |
|  |  |

Note. Reassessing coping items are 4, 5, 6, 8, and 13. Distancing coping items are 1, 2, 7, 14, and 15. Constructive coping items are 3, 9, 10, 11, and 12.

The Center for Epidemiologic Studies Depression Scale (Radloff, 1977)

|  |  |
| --- | --- |
| No. | Item |
|  |  |
| 1 | I was bothered by things that usually don't bother me. |
| 2 | I did not feel like eating; my appetite was poor. |
| 3 | I felt that I could not shake off the blues even with help from my family or friends. |
| 4 | I felt that I was just as good as other people. (R) |
| 5 | I had trouble keeping my mind on what I was doing. |
| 6 | I felt depressed. |
| 7 | I felt that everything I did was an effort. |
| 8 | I felt hopeful about the future. (R) |
| 9 | I thought my life had been a failure. |
| 10 | I felt fearful. |
| 11 | My sleep was restless. |
| 12 | I was happy. (R) |
| 13 | I talked less than usual. |
| 14 | I felt lonely. |
| 15 | People were unfriendly. |
| 16 | I enjoyed life. (R) |
| 17 | I had crying spells. |
| 18 | I felt sad. |
| 19 | I felt that people dislike me. |
| 20 | I could not get "going." |
|  |  |

Note. Reverse-coded items are denoted with (R).
